# Influences on students’ empathy in medical education: an exploratory interview study with medical students in their third and last year

**DOI:** 10.1186/s12909-018-1335-7

**Published:** 2018-10-05

**Authors:** N J Pohontsch, A Stark, M Ehrhardt, T Kötter, M Scherer

**Affiliations:** 1 Department of General Practice / Primary Care, University Medical Center Hamburg-Eppendorf, Hamburg, Germany; 2 Institute for Social Medicine and Epidemiology, University of Lübeck, Lübeck, Germany

**Keywords:** Empathy, Undergraduate students, Qualitative research

## Abstract

**Background:**

Empathy is beneficial for patients and physicians. It facilitates treatment and improves physical and psychosocial outcomes. The therapeutic relevance of empathy emphasizes the need to help medical students develop their empathic abilities. Our study aimed to identify factors which promote or hinder the development and expression of empathy in medical students during the course of their studies.

**Methods:**

We interviewed 24 medical students (six male and six female students in their 6th semester as well as six male and six female students in their final clinical year) using semi-structured interviews. The interviews were recorded, transcribed verbatim and analyzed using Braun & Clarke’s thematic analysis.

**Results:**

We identified four main themes influencing the development and expression of empathy. 1) Course of studies: hands-on-experience, role models, science and theory, and emphasis on the importance of empathy; 2) students: insecurities and lack of routine, increasing professionalism, previous work experiences, professional distance, mood, maturity, and personal level of empathy; 3) patients: “easy” and “difficult” patients including their state of health; and 4) surrounding conditions: time pressure/stress, work environment, and job dissatisfaction.

**Conclusions:**

The development and use of empathy could be promoted by increasing: hands-on-experiences, possibilities to experience the patient’s point of view and offering patient contact early in the curriculum. Students need support in reflecting on their actions, behavior and experiences with patients. Instructors need time and opportunities to reflect on their own communication with and treatment of patients, on their teaching behavior, and on their function as role models for treating patients empathically and preventing stress. Practical experiences should be made less stressful for students. The current changes implemented in some medical school curriculums (e.g., in Germany) seem to go in the right direction by integrating patient contact early on in the curriculum and focusing more on teaching adequate communication and interaction behaviors.

## Background

Communicating with patients is a crucial component of a physician’s daily work. In this respect the ability to be empathic is an important factor for successful physician-patient communication. Physicians’ empathy is beneficial for the patients [e.g. better physical and psychosocial outcomes [1]] and the physicians [e.g. more accurate symptom report and diagnosing [2, 3]].

Definitions of empathy are manifold. They vary from empathy being the “appropriate understanding of another person” [ [4], p. 332] to the ability to understand and mirror patients’ feelings adequately [5] and the intention to help [6]. A recent meta-ethnography identified a certain “conceptual confusion” [ [7], p. 1217] in medical students’ definition of empathy. Some authors see empathy as an emotion [8] or cognitive attribute [4] while others deem it a personality trait [9]. Most authors can consent on empathy having a cognitive component, that means empathy being someone’s’ ability to understand and reflect someone else’s emotions [e.g., [6, 8, 10, 11]]. According to Mercer and Reynolds [12] healthcarers’ empathy is a complex, multidimensional construct including: understanding the patient, reflecting your understanding, checking whether you understood the patient right and acting upon that understanding in a therapeutic way.

Medical students’ empathy has been widely researched, most of the time using self-report measures [for example the Jefferson Scale of Empathy [6]] [13]. There are some critical voices on the reliability of self-report measures of empathy (used in the above mentioned studies) and their reflection of actual differences in bedside behavior [14, 15], but as they are the most easily administered measures they are often used for studies on students’ empathy [15]. Either way, it is undeniable that medical education, role models and patient contact have the potential to influence students’ empathy in a good or bad way, depending on the configurations of these aspects [e.g., [5, 11, 16–21]].

One can assume that medical students‘empathy evolves and changes during their medical education [19, 20, 22, 23]. Previous studies produced contradictory results, showing stable, declining or increasing empathy scores in medical students [15]. Some longitudinal studies show a decrease in empathy throughout medical education [6, 10, 16–18, 20] and students suffering from burn-out symptoms show lower empathy scores [21]. The “hidden curriculum” [24] or the organizational culture [20] may be reasons for this.

In Germany, the “classical” medical curriculum is divided into a preclinical phase of two years, followed by the first medical exam, the clinical phase of another three years followed by the second medical exam and the practical year followed by the third medical exam [25]. In the first two years, students usually don’t have much patient contact. In the clinical phase, students see patients in one- to two-week internships in certain subjects, but the curriculum is still predominated by theoretical input. In the final (practical) year, students work on wards or in practices full-time. Reformed curricula in Germany [e.g. at the Charité (Berlin) since 1999 and at the University Medical Center Hamburg-Eppendorf since 2012] aim at an early and regular patient contact from the beginning of studies on, e.g. by eliminating the boundary between the preclinical and clinical phase [26]. They also include communication and clinical skills courses from the very beginning [27].

Qualitative studies exploring factors that influence empathy development are rare. Eikeland et al. found that among 3rd year medical students acquiring specialized biomedical knowledge was a higher priority than developing soft skills. The additional ubiquitous time-pressure leads to a negative impact on empathic skills [11]. Two studies in Germany [16, 28] created a list of open questions to be answered in writing by medical students about factors promoting or hindering empathy. The authors were able to identify some influencing factors [e.g. prejudices, patient contact, practical skills, patient characteristics, physician-patient-relationship, working conditions, and time pressure [16, 28]] but recommended more elaborate qualitative interview studies in their conclusion [28]. Other authors also conclude the need to identify students’ perspective on aspects of the formal curriculum and the actual practice of medical education that foster or hinder empathy (development) in medical students [8, 29].

International research and recommendations have repeatedly emphasized the importance of helping medical students to develop and increase their empathy [5, 8, 16, 18]. The therapeutic relevance of empathy supports the need to study medical students’ views on and concepts of empathy [7] and help medical students develop and maintain their empathy, and emphasizes the importance of designing curricula to support this development [19, 30]. Given the lack of exploratory studies allowing students to openly express their views on empathy and aspects of the formal curriculums hindering or promoting empathy (development), our study aimed at identifying factors which medical students assume to promote or hinder the development, increase and consolidation of empathic abilities during the course of their studies.

## Methods

This exploratory qualitative interview study with medical students in their 6th semester and students in their final clinical year was conducted at the University Medical Center Hamburg-Eppendorf in Germany. It was funded by the Research Promotion Fund of the Faculty of Medicine (NWF 16/04).

### Participant selection and recruitment

We choose a purposive sampling approach [31] to select interviewees. Some of the abovementioned studies show a decline in students’ empathy during their medical education and it is also reasonable to expect that increased experiences with patients and the clinical environment influence students’ empathy [16, 20]. Gender is also known to influence empathy [8, 19]. Therefore we aimed to interview female and male students from their 6th semester and those in their final year to maximize variations in the students’ accounts on curricular influences on empathy.

A staff member of the deanery invited the eligible students to take part in the study via email. Inclusion criteria: students in their 6th semester (group A) or students in their final year completing their clinical internship (group B). Exclusion criteria: insufficient knowledge of the German language. Due to this recruitment method interviewees and interviewer were unknown to each other before the interview.

Participation in the study was voluntary and non-participation had no negative consequences. Students willing to participate contacted the study team by email or phone, then received written and oral information about the study and provided written, informed consent before the interviews were conducted. Participating students received an allowance of 25€.

### Interview guideline and interview conduction

The interview guideline was developed by NJP and AS based on questions asked and results described in other studies on empathy in the context of medical education [e.g., [11, 19, 21, 28, 29, 32] and sought to identify promoting and hindering factors for empathy. To avoid influencing the students’ account by predefined concepts of empathy we decided to work with students’ subjective definitions of empathy (results will be published elsewhere).

The interview guideline (see Table 1) included the following topics: short introduction of interviewer and study, subjective definition and meaning of empathy in the clinical setting, handling patients’ emotions, reasons for (not) being empathic towards patients, differences between personal and clinical empathy, factors promoting and hindering empathy, ideas for teaching empathy and self-reflection concerning the student’s empathy during the course of studies. In addition we asked students in their clinical internship (group B) to describe the influences of their practical work on their empathy.

**Table 1:** Interview guideline

Medical empathy	
What does it mean from your point of view to be an empathetic doctor?	
Please describe your own empathy in patient contact.	
Empathy with patients	
Which value do you attach to being empathetic with patients?	
How do you handle patients‘emotions and feelings?	
Did you encounter situations in which it was hard for you to understand and cater to a patient’s feelings and views, hence being empathetic? Can you describe these situations?	
In which situations was it easier for you to cater to a patient’s feelings and views?	
Only for students in the final clinical year: Would you say, that your manner of handling patients and catering to their feeling has changed through the experiences you made until now in your final clinical year? Please describe …	
Thinking of your clinical experiences until now, would you say that there is a difference between your empathy in your private life and your empathy with the patients? Please describe …	
Empathy in the course of studies	
What aspects of your studies have helped you to develop empathy with patients?	
What aspects of your studies have hindered you to develop empathy with patients?	
Do you have wishes or suggestions for the future concerning empathy in medical education?	
Has your empathy changed during your course of studies?	
End	
Do you want to add something concerning empathy or empathy in medical education?	

AS conducted all semi-structured qualitative interviews [33, 34] at the Medical University Center Hamburg-Eppendorf between April and June 2016. This method of data collection allows interviewees to talk openly about their experiences while being thematically guided by the interviewer. All interviews were digitally recorded and transcribed verbatim by a trained research assistant following designated transcription rules. Interviews were anonymized during transcription. Following the interviews students were asked to fill out the Jefferson Scale of Physician Empathy [35] and the Saarbrücker Personality Inventory [36] (data not shown here).

### Data analysis

We analyzed the interview data using Braun & Clarke’s thematic analysis [37, 38], a method for identifying, analyzing, and reporting themes within qualitative data. It combines minimal organization with a rich detailed description of the dataset [37]. We chose the semantic approach focusing on the identification of explicit meanings of the data, following a realistic paradigm [39]. This method of analysis includes the following steps: familiarizing yourself with your data, generating initial codes, searching for themes, reviewing themes, defining and naming themes and finally producing the report. AS and NJP familiarized themselves with the data by producing and discussing case summaries for every interviewee. AS conducted the inductive coding and theme development process in close consultation with NJP. To secure the intersubjective comprehensibility and credibility of the analysis [40], the results were presented to and discussed with TK as well as with an interdisciplinary work group for qualitative methods led by NJP. The data was managed using MAXQDA 11 (Verbi GmbH).

### Researcher characteristics

ME is a female MD, board certified in General Medicine, and senior physician for medical education [e.g., [30, 41]]. TK is a male MD; board certified in General Medicine, MSc Public Health and qualified as a professor, with extensive experience in the field of research in students’ health [e.g., [42–45]]. NJP is a female trained psychologist and postdoctorate researcher with comprehensive experience in conducting focus groups and interviews as well as qualitative data analysis [e.g., [42, 46–48]]. AS holds a bachelor degree in physiotherapy and a master degree in health sciences, she is a female researcher with some experience in interviewing and qualitative data analysis [e.g., [49]]. MS is a male full professor of Medicine, board certified in General Medicine, with extensive experience in quantitative and qualitative research [e.g., [30, 50]].

## Results

### Participants

We interviewed 24 students. Interviews lasted between 35 and 90 min (Ø 57 min). For further interviewee characteristics see Table 2. Interviewee IDs were structured to indicate the interviewee’s number and status (Interview_01–12 for students in their 6th semester and Interview PJ_01–12 for students in their final clinical year).

**Table 2:** Interviewee characteristics

Group	Sex (n: female/male)	Ø age (years, min-max)	Ø number of semesters
A	6/6	24 (21-29)	6
B	6/6	29 (24-43)	13

### Factors influencing empathy (and its development) in medical students

Four main themes with a total of 16 subthemes (listed in Table 3) were identified when looking at the factors promoting and hindering empathy.

**Table 3:** Main and subthemes

Main themes	Subthemes
COURSE OF STUDIES	Hands-on-experience
	Role models
	Science and theory
	Importance of empathy
STUDENTS	Insecurity and lack of routine
	Increasing professionalization
	Previous work experiences
	Professional distance
	Mood
	Maturity
	Lack of empathy
PATIENTS	Easy and difficult patients
	State of health
SURROUNDING CONDITIONS	Time pressure/stress
	Work environment
	Job dissatisfaction

### Course of studies

According to the interviewees, different curriculum-related aspects influence empathy development. Students considered (early) hands-on-experiences, contact with real patients and courses preparing them for difficult communication situations as important elements supporting the development of empathy.

*“I always found it helpful when actors played the role of patients. Like in the OSCEs [objective structured clinical examination] in which situations were simulated including a patient history or diagnostic results or something else. I found it helpful to work with patients directly. I find personal patient contact helps to teach empathy in general. Other than that, it’s not one of the main topics in medical school.” (Interview PJ_07, paragraph 112).*

Positive role models, physician or nurse instructors, demonstrate how to cope with stress and treat patients respectfully regardless of the situation, while negative role models can serve as cautionary examples or might have a negative impact on the observer’s empathy.

*“[…] Well, the worst example is Professor V. with his [lecture on XY], he was just sitting there making himself look good, and actually held a very good lecture, but then a patient collapsed in the lecture hall and he didn’t even go to the patient.” […] (Interview PJ_03, paragraph 146).*

*“[…] But that’s why it was always been clear that the worst extreme is inacceptable behaviour. […] I have, as I already mentioned, experienced both. (.) I then took the things I found good and tried to integrate them into my daily work, things that made me think ‘so this is how one should react’, or ‘this is the best method’, or ‘this is how one feels well cared for’.” (Interview PJ_04, paragraph 171–174).*

The strong focus on theoretical knowledge and heavy load of learning matter reduces students’ motivation to engage in bedside teaching sessions, which are usually seen as beneficial for empathy development. Furthermore, a discrepancy exists between the focus on correct medical nomenclature and the need to use common terminology and simplicity to communicate at eyelevel with patients.

*“[…] When one only focusses on the scientific aspect, learning facts by heart aspect, instead of focusing on the human aspect and the person behind the patient, always treating everything as just another case, then one works according to standard procedures. It definitely hinders empathy. […]” (Interview PJ_04, paragraph 172).*

Students reported a strong curricular focus on the importance of empathy in the physician-patient-relationship which supports the development of empathy in contact with patients. In contrast, some faculty members displayed little empathy towards the students which, in turn, hindered students’ empathy.

*“I find the expectations of us students too high, and don’t agree with the way we were treated. […] For example, the expectations in the first four semesters are extremely high. The work load is unbelievable and the entire time I felt as though […] everyone is just expecting something of you but no one in the machinery of medical school actually sees you as a human being, nor is anyone interested in doing so. You have to fulfill the expectations and that’s it. […] I had a very empathic physiology professor, […] he treated me well and I was met with empathy. This is very seldom amongst the teachers in medical school. […]*” *(Interview PJ_03, paragraph 144).*

### Students

Insecurity or a lack of routine and increasing professionalization both influence students’ empathy. As long as the students are insecure about how to communicate with patients, take medical histories and break bad news, it is more difficult for them to be empathic. Insecurity about technical details and a lack of experience in handling the constant lack of time add to these difficulties. While increasing professionalization frees capacities for empathy in daily routines, emotional distancing may decrease empathic behavior.

*“[…] Well the first conversations are always a bit rough, because one always has the guideline in mind: “What do I have to ask next? Medical history questions.” […]. When one […] has learned the guideline, then one can pay more attention to one’s empathy, […]” (Interview_01, paragraph 6).*

*„[…] As soon as one has acquired a certain routine, then I feel as though the empathy automatically becomes less. […] (Interview_05, paragraph 88).*

Some students reported that vocational experiences gained before starting to study medicine helped them to be better prepared for handling patients in an empathic way, especially if they had worked in medical fields (e.g. as paramedics or nurses).

*“[…] (I) am from a therapeutic profession, I already have the knowledge and the experience, both of which I can’t lose anymore and are a good basis for me now. […]*” *(Interview_02, paragraph 74).*

Keeping a professional distance was seen as part of an adequate professional behavior in physicians. Too much empathy might impair the students’ mental wellbeing. Showing empathy while maintaining a certain distance protects the student from an emotional overload in difficult situations, and is seen as a sign of professionalization. Finding the balance between closeness and distance is seen as a developmental task to be achieved by every medical student.

*“[…*] *To react adequately to patients while making sure to maintain a personal/emotional distance and not letting things get so close that one doesn’t know what to do with one’s own emotions. […] so empathetic but also making sure that one doesn’t get too emotionally invested. […]*” *(Interview_02, paragraph 88).*

Students report that the lack of empathy can be temporary or habitual. While sometimes one’s own negative mood hampers empathic behaviour, other students just seem to lack empathic abilities in general. More life experiences and personal maturation seem to be seen as facilitators for the development of empathy and empathic behavior.

*“[…] There is (..) a limit regarding a person‘s emotional capactiy, [empathy can be shown better when one] doesn’t have additional personal stress, three friends with problems and a sick father or something else.”* *(Interview_03, paragraph 78).*

### Patients

Students mentioned different patient characteristics as important for facilitating respectively impeding an empathic relationship. For example, empathic behavior is facilitated by patients being likeable, cooperative, open, sociable, adherent, optimistic, patient, informed and appreciative. Patients showing health-damaging behavior, who ignore medical advice, are unappealing, irrational, thankless, demanding, uninformed, disrespectful, or talk too much as well as those who have extremely differing world views, seem to make empathy more challenging.

“*If I see that someone smoked his entire life, drank a lot of alcohol and now needs a new liver or has lung cancer from smoking, […] I’m not as much on his side as with someone who became ill without being responsible for it.”* *(Interview_PJ_02, paragraph 60).*

The type and degree of an illness also seemed to influence students’ willingness to show empathy. Patients with more serious and complex illnesses are more prone to be treated empathically, because this is seen as helping them to accept their illness or be optimistic concerning the course of their illness. Patients seeming to be scared, reserved, depressed or otherwise mentally sick usually elicited more empathy. Students reported problems showing empathy towards non-responsive patients, e.g. persons with dementia or unconscious/narcotized patients.

*“Usually it’s the patients that have a long medical history or seem a bit scared, or are terrified of a certain diagnostic procedure, where it’s worth the effort to spend a bit more time speaking with the patient and to show a bit more empathy.”* *(Interview_PJ_02, paragraph 30).*

*“[…] I sometimes find it difficult [to have empathy with] patients who suffer from dementia, are bed-ridden, need a lot of care or who no longer communicate with the people around them. I also find that one should also consider how much these people might actually still understand of what one communicates […]”* *(Interview_07, paragraph 48).*

### Surrounding conditions

Students reported that time pressure and stress reduce the empathy of physicians in hospitals. Their own experiences during their medical clerkship, bedside teaching or secondary employment in medical settings support this. Time restrictions require the exertion of technical competencies while supplanting interpersonal competencies. This leads to a “cost-benefit-analysis” of empathic behavior versus fast treatment. Having enough time facilitates empathic behavior, while insufficient staffing, a plethora of duties not requiring patient contact and a high work and stress load lead to less empathy.

*“[…] the physicians‘workloads do not allow for them/ (.) to be empathetic. […] they have so much to do in a day that it’s very difficult to additionally show empathy.”* *(Interview_PJ_01, paragraph 204).*

The work environment also influences the students’ abilities to show empathy. Good communication within the medical team was thought to facilitate empathy by providing relevant information on patients’ problems, while having colleagues talk disparagingly about patients could reduce one’s empathy by creating a biased attitude.

*“[…] Said nurse reported that a patient was just complaining about pain, because she didn’t want to go home before going to rehab. This had such a strong effect, that is what I mean with ‘filter’, of the physicians that no one bothered to properly examine her anymore. […]”* *(Interview_PJ_03, paragraph 60).*

The setting of ward visitations, e.g. unplanned examinations, groups of colleagues in laboratory coats and students’ fear of failing in front of fellow students and senior consultants, hamper empathy. Poor working conditions during clinical internships, e.g. a high work load, a lot of overtime and the excessive expectations of young professionals, but also an underload and doubts about the reasonableness of the health care system can reduce empathic abilities.

*“Yes, well if I, as an employee, consider most of the work I do useless or repetitive and I don’t feel appreciated, useful or rewarded, then of course I’m significantly less positive and, thus, less empathetic.”* *(Interview_PJ_11, paragraph 80).*

*“[…] due to this DRG-system the medical system has become a rotating door, and a patient simply is no longer considered in his/her entirety as a human being […]”* *(Interview_PJ_11, paragraph 48)*

## Discussion

### Main findings

Empathic behavior in medical students seems to be influenced by aspects of their course, individual student and patient characteristics and in the general surrounding conditions. Imparting the high value of empathy on students through communication courses, hands-on-experience and positive role modeling seem to be crucial from the students’ point of view. Creating conditions which allow the unpressurized exercise and exertion of empathy, handling of patients and reflection on these experiences is also considered crucial.

### Strengths and limitations

Our study is the first (German) study to qualitatively explore the opinions of medical students on which factors promote and impede empathy or the development of empathic behavior towards patients. By conducting open qualitative interviews, we were able to explore rather under researched aspects from the point of view of students in the middle and at the end of their medical studies. This study fills a gap indicated by other studies [28].

The major limitation might be that our interviewees come from only one university medical center in Germany. However, some of our results are consistent with results from other German [28, 32] and international studies [7, 11, 24, 29]. Interviewees participated voluntarily in our study, this might have resulted in a self-selection of participants who are exceptionally interested in the topic of empathy. This possible bias cannot be ruled out, but the variation of accounts by the interviewees, which we also tried to maximize by selecting participants by gender and semester, indicates that the participants differed in their experiences with and views on factors hindering and promoting empathy in medical studies.

Despite the self-selection of our participants, they are comparable with samples from other, larger studies concerning their scores on the Jefferson Scale of Physician Empathy [35] and the Saarbrücker Personality Inventory [36] (data not shown).

### Implications of the findings in existing research

Some of our findings, for example the significance of positive role models, time constraints, stress and insecurities, mirror those of other studies conducted in Germany [e.g. [16, 28]] and internationally [e.g. [11, 51, 52]]. Other findings depict new aspects, such as the importance of distance between patients and physicians/students, increasing professionalization and doubts about the reasonableness of the health care system as factors negatively affecting empathy.

Characteristics of the individual patient and/or medical students cannot be directly influenced by changes in the curriculum. The selection of medical students to be accepted for medical studies cannot be solely based on empathic abilities or mental health measures. A lack of patient contact early on in the students’ studies is a barrier to the development of empathy [53]. Increasing chances to gain early experiences with patients and to reflect on one’s own behavior as well as that of the patient could remedy that. Communication training focusing on empathy and the handling of negative emotions, as well as giving students the opportunity to slip into the patient’s role could be helpful [54, 55]. Another kind of practical experience is learning in simulated situations, for example a simulated hospitalization, old age simulations [56] or full scale simulations [57].

Respectful and empathic treatment of students and patients by medical teachers should be strongly encouraged [58] and stress-inducing hierarchies should be leveled [32]. Medical teachers should get the chance to reflect on their own, in order to help increase the much-needed self-reflection in students [59]. This could also support them in their function as positive role models for medical students [53, 58]. Mentoring-relationships, with older students [60] or postgraduate physicians, could support students by counteracting the negative effects of the “hidden curriculum” [24].

A certain degree of emotional distance can serve as self-protection [52], but too much distance results in an indifference towards the patient [51]. Students should be supported in finding the right balance between professional distance and empathy. Seminars teaching strategies on how to handle negative emotions and difficult patient-physician contacts could buffer the negative impact of this kind of stress on empathy and help prevent students’ burn-outs [61–68]. Self-awareness trainings or Balint groups increase empathy and personal development, while reducing stress [28, 68, 69].

Assessments drive what students learn, practice and prioritize. Topics included in examinations will be remembered better. This can be increased by performance feedbacks [70]. One way to counteract the empathy decreasing effects of the formal and ‘hidden’ curriculum and increase the priority put on empathy in the course of students’ studies would be the repeated formal evaluation of the students’ empathic abilities throughout the curriculum (e.g. with simulated patients) [71, 72].

## Conclusions

Reformed curricula promoting early patient contact and including clinical skills and communication courses seem to be a step in the right direction to promote empathy in medical students. Fostering mentoring-relationships and self-reflection in students and teachers, and the integration of evaluations of empathic abilities would also advance empathy development of medical students.
